# Controlling the Spread of Disease in Schools

**DOI:** 10.1371/journal.pone.0029640

**Published:** 2011-12-29

**Authors:** Benjamin J. Ridenhour, Alexis Braun, Thomas Teyrasse, David Goldsman

**Affiliations:** 1 Eck Institute for Global Health, Department of Biological Sciences, University of Notre Dame, Notre Dame, Indiana, United States of America; 2 H. Milton Stewart School of Industrial and Systems Engineering, Georgia Institute of Technology, Atlanta, Georgia, United States of America; University of Zaragoza, Spain

## Abstract

Pandemic and seasonal infectious diseases such as influenza may have serious negative health and economic consequences. Certain non-pharmaceutical intervention strategies – including school closures – can be implemented rapidly as a first line of defense against spread. Such interventions attempt to reduce the effective number of contacts between individuals within a community; yet the efficacy of closing schools to reduce disease transmission is unclear, and closures certainly result in significant economic impacts for caregivers who must stay at home to care for their children. Using individual-based computer simulation models to trace contacts among schoolchildren within a stereotypical school setting, we show how alternative school-based disease interventions have great potential to be as effective as traditional school closures without the corresponding loss of workforce and economic impacts.

## Introduction

Pandemic diseases, such as the 2009 H1N1 influenza pandemic, threaten global health and economics if their spread is unchecked [1]–[5]. The impacts of pandemics have both top-down (imposed government or corporate regulations on individuals) and bottom-up (loss-of-workforce due to individual morbidity and mortality) repercussions [1]–[3], [6], [7]. Therefore, lessening the impact of pandemics via public health interventions is critical. Various non-pharmaceutical intervention strategies – for instance, wearing face masks, increased hand washing, and school closures – are a first line of defense against such threats because they can be implemented rapidly (in contrast to vaccination campaigns, etc.) [8]–[12]. These types of interventions seek to reduce the effective number of contacts between individuals within a community, and have been shown via post hoc analysis to ameliorate the impacts of previous influenza pandemics, e.g., the 1918 Spanish influenza pandemic [13]–[15]. However, the efficacy of closing schools to reduce disease transmission among school-age children is unclear [16]–[18], and school closures result in significant economic impacts because caregivers leave the workforce to care for unattended schoolchildren [1]–[3], [6], [19] – making the decision to close schools in a community controversial. Another complicating factor is that previous models have indicated that the timing of school closings is critical to the disruption of disease dynamics within a community and have typically recommended specific closure durations (e.g., 1–2 weeks) [8]–[11], [16], [20], but implementation of school closures according to the specified timing and duration guidelines is greatly hampered by a community's ability to detect disease prevalence and sustain economic losses.

In light of the potential problems involving school closure, one might ask whether there are reasonable alternatives. Our research uses individual-based computer simulation models to trace contacts among schoolchildren within a stereotypical school setting. In doing so, we show how other alternative school-based disease interventions have great potential to be as effective as traditional school closures, but without the corresponding loss of workforce and undesirable economic impacts.

As it became evident that an H1N1 influenza pandemic was emerging during the spring of 2009, local and national governments around the world started to focus on strategies for mitigating the impact of the looming pandemic. In Mexico, large-scale public health campaigns educated people about protective practices and eventually culminated in an unprecedented closure of government, schools, and businesses for a week to disrupt transmission of the disease [21]. Within the United States, similar, but less drastic, non-pharmaceutical disease interventions were being considered and implemented. By the end of April, schools in the Dallas–Fort Worth region of Texas (as well as elsewhere within the U.S.) were closing as a measure to control the spread of the pandemic influenza strain [22]. Originally, the U.S. Centers for Disease Control and Prevention (CDC) recommended, as part of its guidance for non-pharmaceutical interventions, that schools close for a period of two weeks; by 5 May 2009, however, the CDC revised those recommendations to one of merely isolating sick children. All told, 726 schools closed for various periods of time within the U.S. during this early-pandemic period [13].

School closure policies and policy changes met with criticism from the public within the U.S. due to the associated loss of productivity. Research has indicated that school closure could have an economic impact of up to £0.2–£1.2 billion per week in the U.K. (based on 2005 data and uncorrected for inflation) and thus is potentially a costly intervention for countries to impose [2]. Furthermore, although research is still being conducted on the issue, it is unclear how beneficial school closure is with regard to slowing a pandemic on local and national scales [8]–[10], [16]–[18], [20], [22]. In fact, the efficacy of school closure at preventing disease spread is unknown in general because school closure is often confounded by seasonal holidays, bans on public places, or increased circulation of children outside of school [14], [23], [24]. Thus, school closure represents a potentially costly intervention with unknown effects.

**Figure 1:**
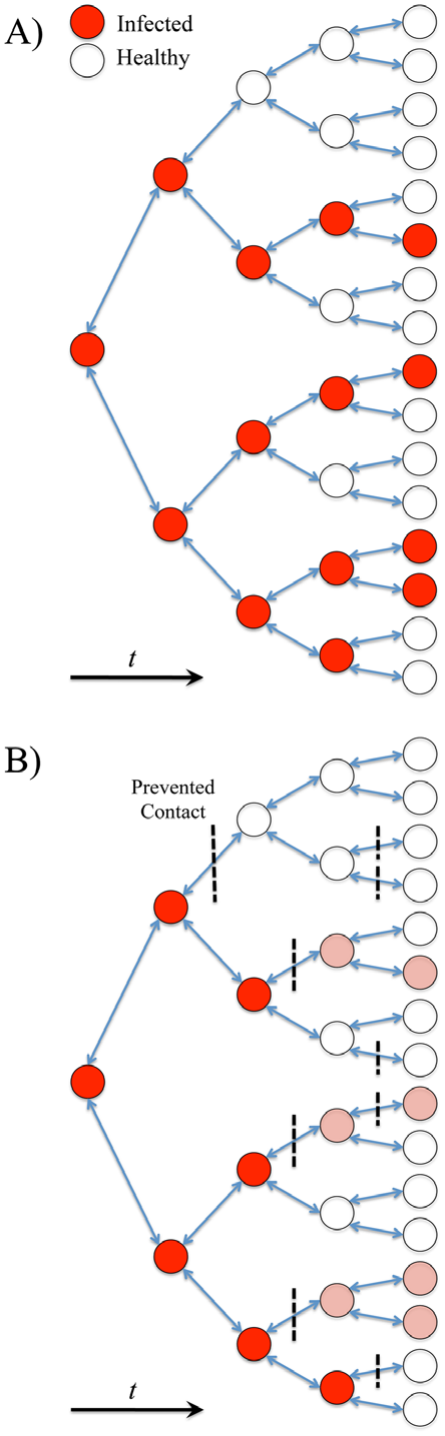
Basic disease transmission. When infected individuals (red circles) contact other individuals (shown as arrows), those individuals become infected with some probability based on the transmissibility of the disease weighted by the duration of the contact between the two individuals; conversely, some individuals are not infected during a contact (empty circles). School closure is aimed at disrupting/eliminating contacts between children, who are often the most effective disease transmitters. Panel A shows undisturbed transmission in a population, while Panel B shows the reduction in transmission in a similar population where contacts have been blocked/avoided due to an intervention.

The spread of a disease can be viewed as a chain (Fig. 1) or as an “infection tree.” Transmission between individuals requires that (1) individuals come into contact and (2) as a result of that contact, an individual becomes infected. Contacts between individuals may be direct or indirect, for example, via non-sterile surfaces. Because transmission among school children is considered to be a primary mode of disease propagation [10], [11], [25], school closure is an appealing intervention – thereby suspending all contacts that would occur amongst children within schools. Closing schools may also protect one of the most vulnerable age classes within the population. For example, the 2009 H1N1 influenza pandemic disproportionately affected individuals under the age of 65 in terms of mortality and morbidity [26], and children under the age of 18 may have been subject to the highest attack rates [27].

Many recent models of school closure utilize a synthetic community structure where students who are dismissed from school are still active within the community [10], [11]. Contrary to what might be expected, the loss in contacts in a school setting does not necessarily lead to an overall loss in contacts within a community, and, in turn, a reduction in disease transmission. These non-intuitive results of school closure are sometimes referred to as “perverse effects.” Unfortunately, little-to-no real-world data currently exist on the frequency of in-school contacts [28], [29] versus in-community contacts when schools are closed, but it is generally assumed that there is a net reduction in contacts.

Regardless of the presumed changes in contact rates, the messages of models of school closure for influenza are nearly unanimous: If the number of contacts among school children is reduced sufficiently early within a pandemic peak disease, then incidence rates are reduced and the time to peak incidence can be delayed [8]–[11], [14], [16]–[18], [20], [23], [24]. These benefits coupled with ease of implementation make the closing of schools particularly tempting – despite the negative ramifications – especially when compared with other interventions such as vaccination where the vaccine's effectiveness is unknown and its production takes many months.

## Analysis

**Figure 2:**
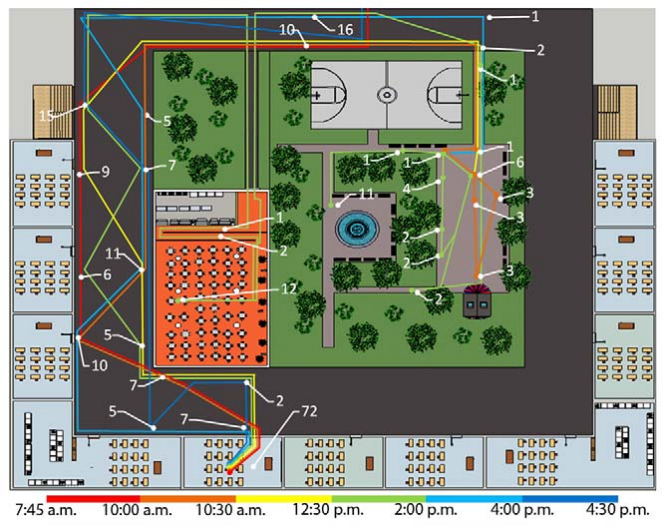
Layout of school's lower level and single student example. The figure shows the general layout of the school. The entrance to the building is at the top-center of the figure. The hallway (dark gray) encircles the lunchroom (orange) and the schoolyard. Classrooms sit on the exterior walls of the building. Stairs (tan) allow students to go to the upper level of the school. The upper level has the identical layout but without the lunch and recess spaces. Colored lines indicate the paths traveled by one student during a particular realization of the simulation. Line colors indicate the time of day the path was traveled. The numbered white lines indicate how many contacts occurred in a particular school area.

Because of the uncertainty of vaccine production, delivery, and effectiveness, and the costliness of even short-duration school closures, we investigated alternatives to school closure that may achieve similar beneficial results. To this end, we simulated the number of contacts occurring among individuals within a typical school setting (based on a U.S. school structure) using the discrete-event simulation software Arena from Rockwell Automation [30]. Our archetypal school houses 352 students based in 22 classrooms, each of which is home to 16 students (Fig. 2). The school building consists of two floors with a lunchroom and a schoolyard; the schoolyard is used during recess periods. A central entryway to the building and hallways allow student movement into and around the school. Our simulation monitored student movement and contacts; every contact occurring during a school day was written to a Microsoft Access database with a time stamp, the individuals involved, and the location. We analyzed the simulation results after the completion of 30 replications of each intervention strategy.

**Figure 3:**
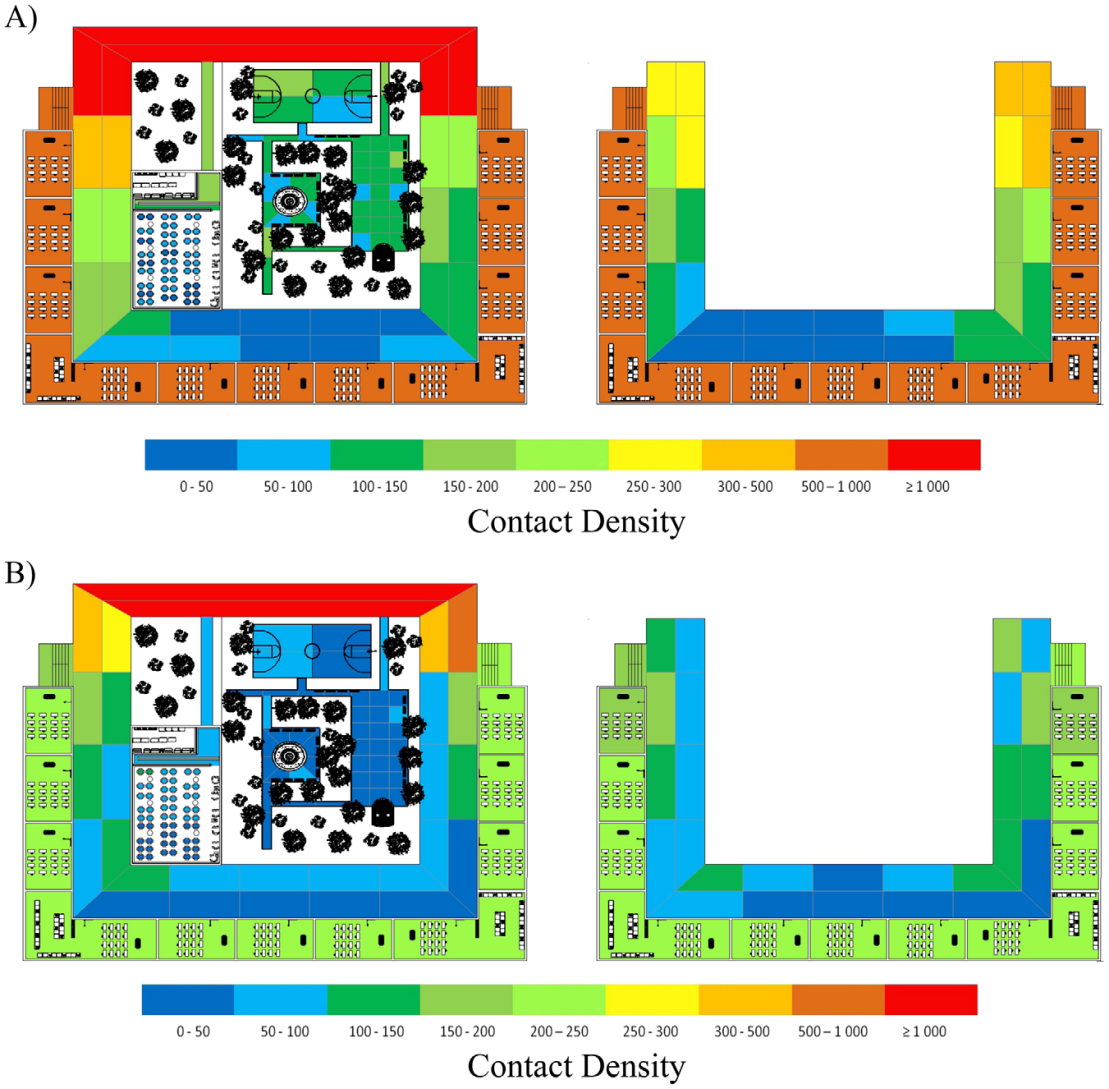
A heat diagram showing total contacts occurring in each school area during a simulated day. Panel A depicts the number of contacts that occurred during the baseline (B) set of simulations; Panel B depicts the number of contacts that occurred during the most-effective intervention (AAI). The left-hand column is the lower level of the school and the right-hand column the top level. Unsurprisingly, the highest density of contacts occurred in the entryway of the building (top-center lower level). Without interventions, schoolrooms have a relatively high number of contacts; with the AAI interventions, the relative contribution of classrooms is greatly diminished. Likewise, contacts occurring in other shared spaces (lunchroom and schoolyard) were also drastically reduced.

In our baseline model, students follow this schedule. Students arrive at school between 7:45–7:50 a.m., and proceed to their classrooms via hallways and/or a staircase by 8:00 a.m. The hallways are divided into 56 different zones (visible in Fig. 3). If hallway movement is not constrained, individuals move haphazardly along the network via a series of Bernoulli random variables. Students stay in their respective classrooms from 8:00–10:00 a.m., at which point they walk to the schoolyard for recess (30 minutes). In the baseline case, students cluster randomly into the 44 schoolyard zones, and students move between zones after random-length stays – a mixture of lengths that are 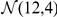, 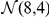, and 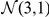 minutes, depending on how much time is left in the recess period, where 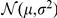 denotes a normally distributed random variable with a mean of 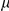 and a variance of 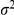. The Arena software does not allow the generation of negative times; thus, for all random time intervals, if any negative random numbers are generated, they are then truncated to zero. Students return to class until lunch at 12:30 p.m. At lunch, students enter a queue to receive lunch and afterward randomly sit at one of the 28 lunch tables (8 places per table). The time for one of three parallel attendants to serve a child after he reaches the front of the lunchroom queue is 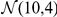 seconds. Once a child sits at a table, the time spent eating is 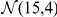 minutes. After eating, students go to the schoolyard to play until 2:00 p.m. Afterward, classes resume until the completion of the school day at 4:00 p.m. A video of a realization of the process is available at http://www.isye.gatech.edu/~sman/speedup.wmv.

Travel times are a function of the distance traveled; travel times and paths both involve randomness. For example, at the beginning of the day, a student must travel from the entrance of the school to his or her classroom. In order to do so, the student moves into new hallway zones in the direction of their classroom. The new zone is chosen uniformly at random from the available adjacent zones in the direction of travel, and the duration spent in that zone is dependent on a student's walking speed (Fig. 2). Students are randomly assigned walking speeds that are 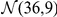 meters per minute distributed (i.e., roughly 1.5 miles per hour).

Contacts are counted separately for each type of school area. Within classrooms, a contact may occur when students occupy adjacent seats. Moreover, students may move within the classroom, so that additional contacts are possible. Contact rates are inversely related to the distance between seated students. For instance, under the baseline case scenario, students one, two, or three seats away from each other have a 90%, 70%, or 50% chance of contact, respectively. Contacts are allowed once an hour. Under a no-movement constraint imposed on classrooms, contact probabilities decrease to 50%, 25%, or 10%, respectively. Adjacency also determines contacts while seated in the lunchroom or in the lunchroom queue as well. For example, students seated at the same table will contact each other with certainty, while students in the serving queue have a 100% chance of contact with adjacent students. Students in the same schoolyard or hallway zone may contact each other with probabilities of 20% and 15%, respectively.

We studied the effects of a variety of interventions that we believed would provide effective reductions of contact rates among children and were relatively non-disruptive to the functioning of the school. The interventions were as follows:

1. Baseline (B): The children can move about the entire school freely on their way to their scheduled rooms, sit anywhere they want in the classrooms, and go wherever and with whomever they want in the schoolyard and in the lunchroom. Every child has the same schedule.
2. Hall Restriction (HR): The children must stay in a defined walking area between their classrooms, the lunchroom, and the schoolyard (typically, the right-hand side of any hall as they travel). There are no restrictions while in the classroom, lunchroom, or schoolyard.
3. Classroom Restriction (CR): The children must remain seated while in their classroom. There are no hall, lunchroom, or schoolyard restrictions.
4. Schoolyard Restriction (SR): Each child stays is a randomly specified schoolyard area (with children from random classrooms). There are no hall, classroom, or lunchroom restrictions.
5. SR with Classmates (SRC): The children stay in a classroom-specific schoolyard area with their classmates. There are no hall, classroom, or lunchroom restrictions.
6. Lunchroom Restriction (LR): The children can eat only with their classmates. There are no hall, classroom, or schoolyard restrictions.
7. Different Schedules (DS): Each classroom follows one of three different schedules put forth by the school: the current schedule, a shift of 45 minutes, and a shift of 90 minutes. Other than that, the children can move throughout the entire school freely, sit anywhere they want in the classrooms, and go wherever and with whomever they want in the schoolyard and lunchroom.
8. All interventions (AI): HR+CR+SRC+LR+DS.
9. Almost All Interventions (AAI): HR+CR+SR+DS.

Although our primary interest in these simulations was to observe the possible reduction in contact counts, we performed a post hoc analysis to examine the influence of the proposed interventions on disease transmission. In order to do this, we utilized the contact data stored within the resulting Microsoft Access databases. We assumed that five infectious individuals entered the school at the beginning of the day. Contacts and the place of contact with these infectious individuals were tallied during the school day. Because duration of contacts plays a role in disease transmission [31], [32], we weighted contacts as follows: contacts lasted 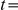
10, 5, 3, and 1 minutes in classrooms, the lunchroom, the schoolyard, and hallways, respectively. Based on total contact duration, the probability of escaping infection 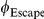 was calculated as 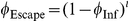 where 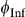 is the probability of transmitting the disease per minute of contact; we examined values of 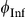 ranging from 0.01 to 0.3. A Bernoulli trial based on 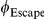 then determined if an individual became infected.

## Results and Discussion

We simulated 30 independent replications of each intervention under the various scenarios. Besides graphically animating the movement of students, we were able to monitor the build-up of queues as well as the utilization of various resources (e.g., seats in a classroom). The multiple replications allowed us to establish statistically valid differences among the various intervention strategies with respect to minimizing the expected number of contacts between children. Table 1 presents the means and standard deviations for the number of contacts counted during the simulation. Here we report on the total contacts among students, decomposed into the various areas of the school, under the intervention strategies outlined above.

**Table 1:** Representative simulation results for total number of contacts among students.

Intervention	B	HR	CR	SR	SRC	LR	DS	AI	AAI
Schoolyard	5,648	5,701	5,648	2,147	2070	5,544	1172	1,362	492
	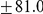	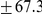	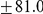	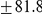	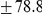	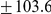	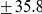	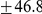	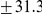
Classroom	11,407	11,413	4,751	11,402	11,415	11,403	11,384	4,736	4,761
	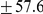	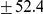	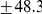	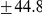	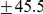	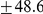	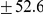	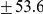	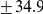
Hall	18,948	16,318	18,948	18,874	18,940	18,842	10,096	8,842	8,906
	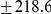	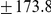	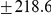	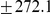	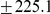	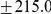	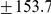	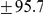	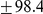
Lunchroom	1,977	1,893	1,977	1,981	1,976	1,953	895	1,591	790
	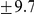	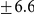	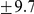	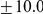	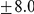	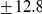	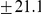	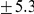	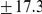
Total Contacts	*37,980*	35,325	31,324	34,404	34,401	37,742	23,547	*16,531*	*14,949*
	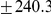	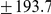	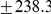	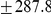	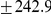	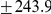	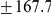	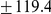	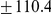

The abbreviations for the different intervention types can be found in the text. Contact counts were tabulated separately for different areas of the school. Results are based on 30 replications of the simulation per intervention; values given are the means 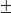 standard deviations.

Generally speaking, the more interventions we imposed, the lower the total number of contacts. The table shows that interventions targeted at specific locations clearly reduced contacts in that location (e.g., schoolyard contacts dropped from 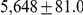 to 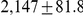 with intervention SR). Each student contacted 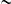
92 other students in the baseline case (B); under the best-case test scenario (AAI), this dropped to 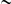
41 contacts per student. Comparing the AI and AAI interventions, it is clear that intervention strategies are not strictly additive in nature. Indeed, there are some interesting anomalies which aptly demonstrate that one must take second-order effects into account when considering policy decisions. Most notably, the AAI set of interventions, in which the LR intervention is turned off and the SRC intervention is partially turned off, actually results in fewer contacts than the AI set (Table 1). This follows because the AI interventions force children to stay amongst their classmates, resulting in more intra-class contacts, while the AAI interventions allow students to spread out less densely in the lunchroom and schoolyard areas, resulting in fewer contacts (Fig. 3).

While our simulation efforts have been motivated by school closures in response to influenza, we have not specifically included influenza disease transmission as part of our model for a number of reasons: First, it is not clear exactly how influenza is transmitted (e.g., droplet versus aerosol) and what an “effective” contact is (i.e., contacts resulting in disease transmission) [33], [34]. Second, school closures are not limited to being used as an intervention for influenza, so our results apply to other directly transmitted infectious diseases for which school closure might be used as an intervention. Third, reducing contact rates should be highly correlated with reducing transmission (as supported by our post-hoc transmission analysis); as discussed earlier, however, there may exist important second-order effects that reduce this correlation. Finally, typical school closure models implicitly use the *identical* mechanism to reduce disease burden, i.e., by stopping contacts.

**Figure 4:**
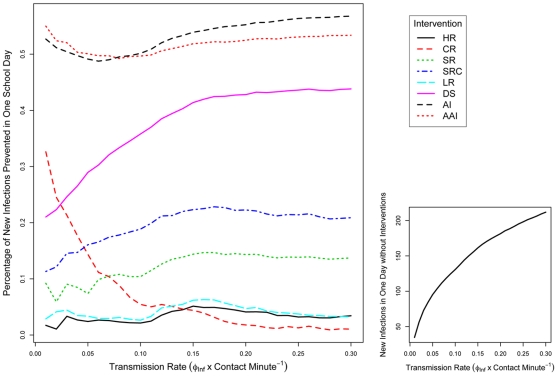
The relative decrease in disease transmission for the proposed intervention strategies. The probability of transmitting a disease per minute was allowed to range between 0.01 to 0.3 for all 8 interventions. The percentage of new infections prevented in one day (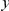-axis) is the relative drop in infected individuals compared to the baseline run (i.e., 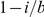, where 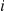 and 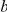 are new infections in the intervention and the baseline simulations, respectively). For the baseline simulations, the total number of new infections depending on the probability of infection is shown on the bottom right.

To verify that reducing contact counts produces a correlated drop in disease transmission, we assessed the number of *potential* transmission events by considering what would happen if five infectious children entered the school (Fig. 4). Transmission events were strictly based on assumed contact durations for given locales and an assumed probability of infection per unit time (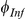 ranged from 0.01 to 0.3; see Analysis). This simple version of disease transmission revealed several interesting results: Using all proposed interventions (AI) outperformed the “almost all” (AAI) intervention strategy despite having a larger contact count. This suggests that AI reduced the number of unique contacts between individuals in comparison to AAI and is therefore perhaps the best intervention option. Similarly, the SR and SRC interventions outperformed expectations based on raw contact counts; this effect is probably driven by the high degree of mixing – which produces novel contacts – that we assumed would occur during school recess. Another unexpected result of our transmission analysis was that the classroom restrictions (CR) can be quite effective, and, in particular, was the best single intervention (i.e., excluding the AI and AAI interventions) at lower infection probabilities. This result occurs because of the extended duration of classroom contacts between students; when the infection probability per minute is higher, raw contact counts become more important, and the CR intervention no longer performs as well. At higher transmission rates, employing staggered school schedules (DS intervention), is the best single intervention (as expected).

We chose a broad range of transmission rates for our analysis in order to maintain generality. Little empirical information exists on the probability of transmitting during a direct contact; such rates could possibly be inferred, however, from estimates of the basic reproductive number (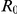) of a disease. For influenza, 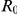 varies seasonally but could potently encompass values from around 1 (e.g., an early estimate of 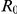 for the 2009 pH1N1 pandemic was 1.2 [35]) to nearly 4 for the 1918 pandemic [36]. These would indicate transmission rates more in line with the rates shown on the left-hand side of Fig. 4. Along similar lines of reasoning, the reduction in transmission shown in Fig. 4 gives an idea of the expected reduction in 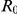 (e.g., if an intervention prevents 30% of new infections, then the intervention, by definition, leads to a 30% reduction in 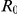). Thus, interventions such as AI and AAI might be necessary for diseases with an 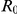 over 2; on the other hand, the less drastic CR intervention might suffice in cases where 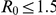 – which is what we would typically expect for influenza.

Clearly, it is necessary to empirically assess the results of our simulations. Critical features of this would be to actually determine the number and duration of contacts that occur in various locales at schools. Furthermore, the contact structure is likely to be variable depending on the type of school under consideration (e.g., elementary vs. secondary schools). As discussed in the previous paragraph, if public health officials were to employ only a single intervention (and not combinations of interventions), then knowing the transmission rate becomes pivotal in choosing the appropriate intervention (Fig. 4). We feel that most of the chosen interventions should be readily implementable in a typical school. Only one of the proposed interventions (DS) actually alters the duration of the school day, and the rest essentially require organization of a student body and enforcement by school officials – which may be no trivial task. Plans are underway to perform all of the above empirical assessments in a U.S. elementary school.

We have shown that alternatives to complete school closure can significantly reduce contacts in a simulated school setting. Use of such alternative methods could allow school and public health officials to freely impose restrictions in schools without the disruptive consequences (social, economic, and educational) of traditional closure strategies. This also allows officials to apply restrictions for longer periods of time, thus increasing the likelihood of successful intervention (compared to brief closures which must be precisely timed to have any benefit). As mentioned above, relying strictly on the simulation results presented here would be overly optimistic; thus plans for future research include empirically examining contact rates in schools [28], [29], the observed effects of interventions such as the ones proposed here, and more in-depth simulation that includes complex school structures and disease-specific transmission.

By utilizing our proposed alternative school-based interventions, economic losses can be minimized, thereby reducing concerns regarding timing and duration of these interventions. In any case, it is essential for policy-making bodies (public or private) to understand the options for and utilization of novel pandemic interventions to minimize impacts associated with future pandemics.
